# Supplementary material for: Visualization of ferroaxial domains in an order-disorder type ferroaxial crystal
Source: Nat Commun. 2020 Sep 11;11:4582. doi: 10.1038/s41467-020-18408-6 (PMC7486364; doi:10.1038/s41467-020-18408-6)
Supplement: Supplementary file 1 — Supplementary Information [file 41467_2020_18408_MOESM1_ESM.pdf]

Supplementary Information

Visualization of ferroaxial domains in an order-disorder type ferroaxial crystal

Hayashida *et al.*

Supplementary Note 1 | Simulation of convergent-beam electron diffraction

Supplementary Figure 1a shows a schematic diagram of formation of a convergent-beam electron diffraction (CBED) pattern. A nanometer-sized local specimen area is illuminated by a conical convergent electron probe. According to the convergence angle of the incident electron probe, diffraction disks are produced instead of diffraction spots in conventional selected-area electron diffraction patterns. Zeroth-order Laue zone (ZOLZ) reflections are seen in the center of the CBED pattern and higher-order Laue zone (HOLZ) reflections appear as a ring surrounding the ZOLZ reflections. Supplementary Figure 1b schematically shows reciprocal lattice points, Ewald spheres corresponding to a convergent beam illumination and their resulting CBED pattern. It should be noted that the HOLZ reflections provide three-dimensional information on the reciprocal lattice, which is essential to distinguish the ferroaxial domains.

The CBED disks exhibit intensity distributions according to the changes of diffraction condition (two-dimensional rocking curves). In Supplementary Figure 1a, each point in the 000 CBED disk corresponds to a different incident-beam direction and is related to conjugate points by reciprocal lattice vectors, as depicted by a small rectangle in each CBED disk. Theoretical intensities of CBED patterns can be obtained by the Bloch-wave dynamical theory of electron diffraction[1]. Eigen states (Bloch wave) in a crystal potential can be obtained by solving the Schroedinger's equation, or diagonalization of the structure matrix[2,3]. Excitation amplitudes of the Bloch waves are determined by the boundary condition at the upper surface of the specimen. The Bloch wave simulations of the CBED patterns in Fig. 3 of the main text were performed with a software MBFIT[4,5].

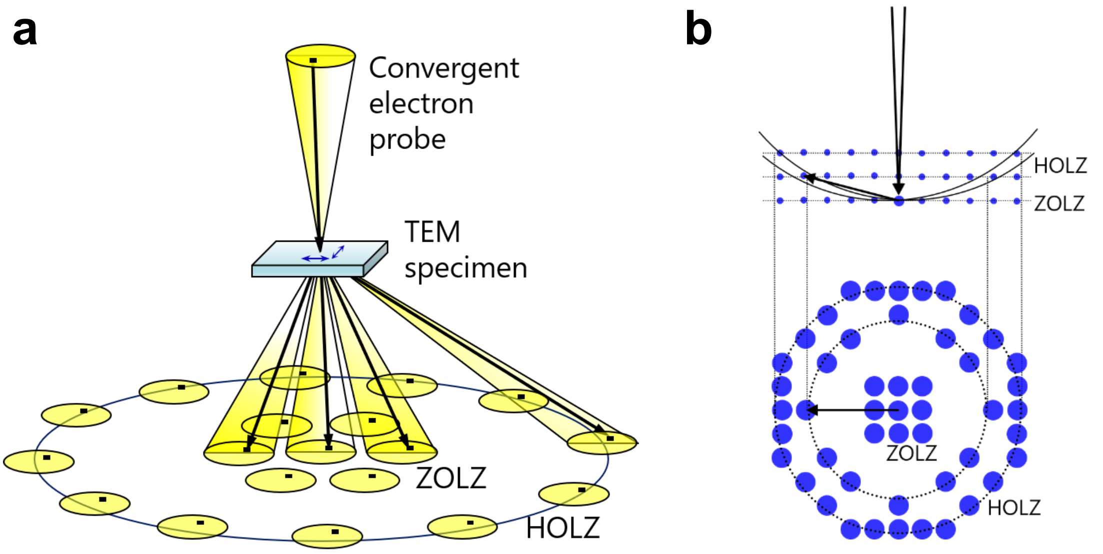

Supplementary Figure 1 | Schematic diagrams of the CBED method. a, Formation of a CBED pattern. **b**, Reciprocal lattice, Ewald spheres, and resulting CBED pattern.

Supplementary Note 2 | Linear electrogyration tensor of point group $\bar{3}$

The linear electrogyration (EG) coefficient γ_{ijk} transforms as an axial third rank tensor. Applying Neumann's Principle to the third rank axial tensor, the linear EG matrices for the respective point groups are obtained. Supplementary Table 1 shows the linear EG matrix of point group $\bar{3}$ (ferroaxial phase of $NiTiO_3$). The γ_{333} component represents liner EG when an applied electric field **E** and a direction of light propagation **k** are both parallel to the c axis (hexagonal setting). Note that the c axis is parallel to an electric toroidal moment **A** in point group $\bar{3}$.

Supplementary Table 1 | Linear electrogyration matrix of point group $\bar{3}$ (orthogonal basis).

γ_{ijk}		k		
		1	2	3
ij	11	γ_{111}	$-\gamma_{222}$	γ_{113}
	12	$-\gamma_{222}$	$-\gamma_{111}$	0
	13	γ_{131}	γ_{132}	0
	21	$-\gamma_{222}$	$-\gamma_{111}$	0
	22	$-\gamma_{111}$	γ_{222}	γ_{113}
	23	$-\gamma_{132}$	γ_{131}	0
	31	γ_{131}	γ_{132}	0
	32	$-\gamma_{132}$	γ_{131}	0
	33	0	0	γ_{333}

This table was made based on Bilbao Crystallographic Server[6–9]. The component γ_{333} measured in this study on $NiTiO_3$ is highlighted in yellow. The orthogonal basis ($\mathbf{a}_\mathrm{o}, \mathbf{b}_\mathrm{o}$, and $\mathbf{c}_\mathrm{o}$) is obtained from the hexagonal basis ($\mathbf{a}_\mathrm{h}, \mathbf{b}_\mathrm{h}$, and $\mathbf{c}_\mathrm{h}$) according to the relations, $\mathbf{a}_\mathrm{o} \parallel \mathbf{a}_\mathrm{h}, \mathbf{b}_\mathrm{o} \parallel \mathbf{a}_\mathrm{h} \times \mathbf{c}_\mathrm{h}$ and $\mathbf{c}_\mathrm{o} \parallel \mathbf{c}_\mathrm{h}$.

For a mirror operation [mirror plane || (110)]

$$\sigma_\mathrm{v} = \begin{bmatrix} a_{11} & a_{12} & a_{13} \\ a_{21} & a_{22} & a_{23} \\ a_{31} & a_{32} & a_{33} \end{bmatrix} = \begin{bmatrix} \frac{1}{2} & -\frac{\sqrt{3}}{2} & 0 \\ -\frac{\sqrt{3}}{2} & -\frac{1}{2} & 0 \\ 0 & 0 & 1 \end{bmatrix} \tag{1}$$

which connects two opposite ferroaxial states (A+ and A–), the third-rank axial tensor γ_{333} is rewritten as

$$\gamma'_{333} = -a_{3i}a_{3j}a_{3k}\gamma_{ijk} = -(1)(1)(1)\gamma_{333} = -\gamma_{333}. \tag{2}$$

This means that the sign of γ_{333}, i.e., the direction of electric-field-induced optical rotation in the configuration of $\mathbf{E} \parallel \mathbf{k} \parallel \mathbf{A}$, depends on that of $\mathbf{A}$.

Supplementary Note 3 | Measurement principle of electrogyration

Here we show how to measure EG, i.e., induction of optical rotation by applying an electric field. A centrosymmetric pyroaxial crystal with the widest faces perpendicular to electric toroidal moment $\mathbf{A}$ is prepared. Indium/tin-oxide (ITO) transparent electrodes are spattered on the widest faces to allow the application of an electric field between the electrodes. The crystal is placed between a polarizer and an analyzer, as depicted in Fig. 4a of the main text. When the angle between the orientations of the polarizer and the analyzer (θ) is set at $\theta = \pm 45°$, the intensity of the transmitted light is expressed as

$$I = \frac{I_0}{2}[\cos\{2(\pm 45° - \phi)\} + 1]$$

$$= \frac{I_0}{2}\{\pm\sin(2\phi) + 1\} \tag{3}$$

where I_0 is the intensity of the transmitted light without the analyzer, and ϕ [deg] is the rotation angle of the polarization plane induced by an applied electric field E [V m^{-1}]. Because the EG effect is usually small, it can be assumed that $\sin(2\phi) \approx 2\phi$ and Supplementary Equation 3 is rewritten as

$$I \approx I_0\left(\pm\phi + \frac{1}{2}\right)$$

$$= I_0\left(\pm\alpha V + \frac{1}{2}\right), \tag{4}$$

where α [deg V^{-1}] is rotation angle of the polarization plane per applied voltage V [V] and represents the magnitude of the linear EG effect. The difference between I obtained in positive and negative applied voltages divided by their average is expressed as

$$\frac{\Delta T}{T} = \frac{I_0\left(\pm\alpha(+V) + \frac{1}{2}\right) - I_0\left(\pm\alpha(-V) + \frac{1}{2}\right)}{\left\{I_0\left(\pm\alpha(+V) + \frac{1}{2}\right) + I_0\left(\pm\alpha(-V) + \frac{1}{2}\right)\right\}/2}$$

$$= \pm 4\alpha V \tag{5}$$

Thus, EG can be readily obtained by measuring the intensity of transmitted light in the polarization configurations of $\theta = \pm 45°$ at applied voltages $\pm V$. Because the sign of α depends on that of $\mathbf{A}$, a spatial map of $\Delta T/T$ is nothing more nor less than that of ferroaxial domains. It is also noted that $\Delta T/T$ will be opposite in sign in the polarization configurations between $\theta = +45°$ and $-45°$. Therefore, domain contrasts will get reversed by changing the polarization configuration from $\theta = +45°$ to $-45°$. Such a contrast reversal confirms that the obtained contrasts are due to the electric-field-induced change in optical rotation, but not that in optical absorption. If the origin of $\Delta T/T$ stems from an electric-field-induced change in absorption, $\Delta T/T$ is independent of the sign of θ.

Supplementary Note 4 | Electrogyration measurement of a reference material $PbWO_4$

To check the validity of our measurement systems, we measured EG of a reference material, $PbWO_4$ whose EG is reported in ref.[10]. $PbWO_4$ belongs to point group $4/m$ which is one of the centrosymmetric pyroaxial groups and is likely to have a single ferroaxial domain state because it has no ferroaxial transition. We used a single crystal of $PbWO_4$ with the widest faces normal to the c axis and the thickness of 0.5mm (MTI Corp.). To form transparent electrodes which allow the application of a voltage parallel to the c axis, ITO was spattered onto the widest faces. For EG measurements, an electric field is applied along the direction of light propagation which is parallel to the c axis ($\parallel$ **A**). In this configuration, EG corresponding to the γ_{333} component is obtained. The spatial distribution of EG in this crystal was visualized by the difference image-sensing technique (see Methods of the main text).

Supplementary Figures 2b-e show the two-dimensional maps of $\Delta T/T$, i.e., spatial distributions of EG, in the same area (Supplementary Figure 2a) of the $PbWO_4$ crystal in various applied voltages. The data of Supplementary Figures 2b-d were taken in the polarization configuration at $\theta = +45°$ while those of Supplementary Figure 2e were at $-45°$. As we expected, the distribution of $\Delta T/T$ is uniform in the whole observation area for all the figures. This means that the observation area consists of a single ferroaxial domain. In addition, overall colors of the maps monotonically change with increasing the magnitude of the applied voltage (Supplementary Figures 2b-d). To quantify the magnitude of EG, we calculated the average of $\Delta T/T$ in all the pixels of the observation area for each figure and took its applied voltage dependence. Supplementary Figure 2f displays the result, which shows that the magnitude of $\Delta T/T$, i.e., the magnitude of EG, is proportional to applied voltage. The linear dependence of $\Delta T/T$ on the applied voltage confirms that the observed EG is ascribed to the linear effect. Furthermore, the sign of $\Delta T/T$ is reversed by switching the polarization configuration from $\theta = +45°$ to $-45°$ (compare Supplementary Figure 2d and Supplementary Figure 2e), which means that the observed $\Delta T/T$ is due to electric-field-induced change in optical rotation but not to that in absorption. The magnitude of EG calculated by using the average of $\Delta T/T$ of Supplementary Figure 2d ($V = \pm 100$ V, $\theta = +45°$) is $\alpha = (6.0 \pm 1.0) \times 10^{-5}$ deg V^{-1}. The error was calculated from the standard deviation of $\Delta T/T$. This α value is in good agreement with that obtained with our lock-in measurement (see Supplementary Note 6) (5.7×10^{-5} deg V^{-1}), though the wavelengths of these two measurements were slightly different. These two α values we measured are not much different from that reported in ref. [10] (9×10^{-5} deg V^{-1} for $\lambda = 630$ nm).

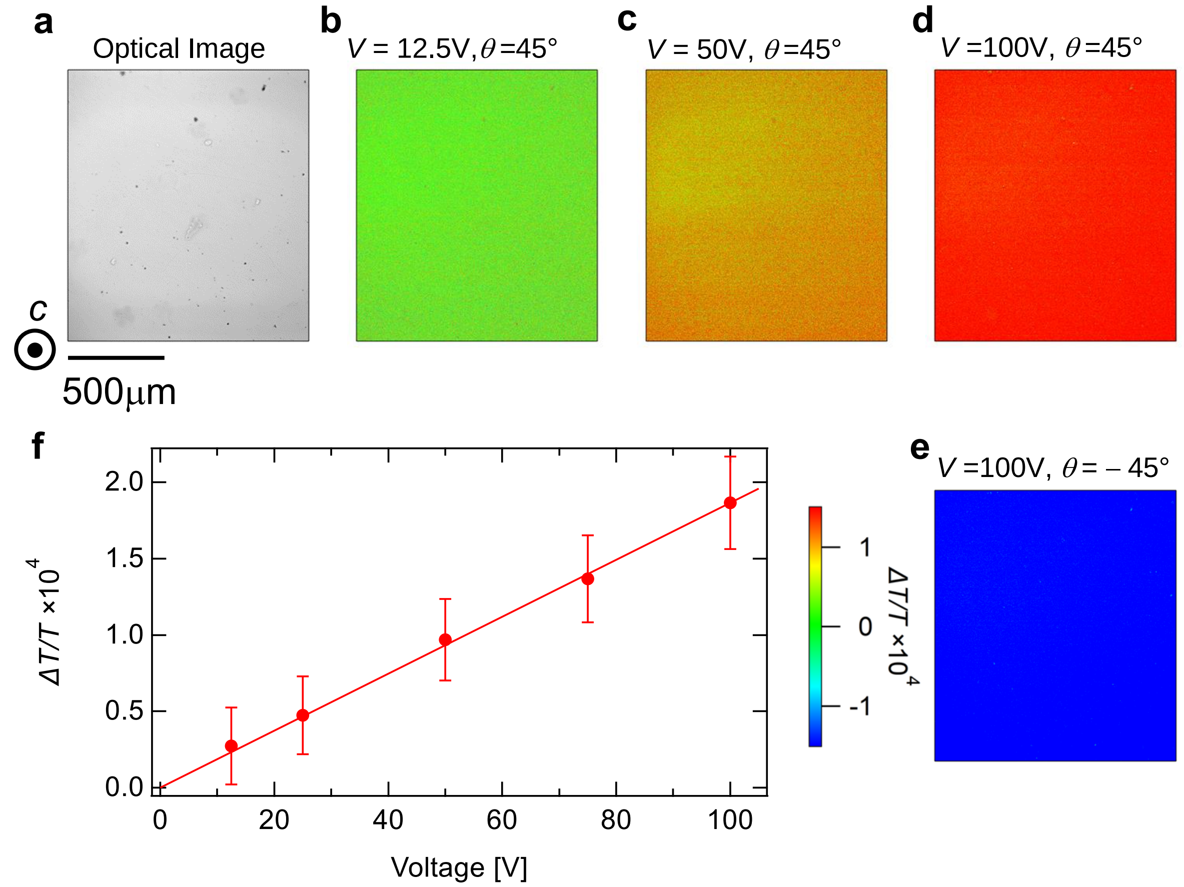

Supplementary Figure 2 | Applied voltage dependence of the intensity map of EG in $PbWO_4$. a, Transmission optical microscopy image with the incidence of unpolarized light along the *c* axis (Scale bar: 500 μm). **b-e**, Two dimensional maps of $\Delta T/T$, i.e., the intensity maps of electrogyration, at the same area as panel **a**. A 3×3 median filter was applied to the raw images. The polarization configuration was set at (**b-d**) $\theta = +45°$ and (**e**) $-45°$. A $\Delta T/T$ color scale is applied to the images in panels **b-e**.The applied voltage V was (**b**) $\pm$12.5 V, (**c**) $\pm$50 V, and (**d**,**e**) $\pm$100 V. **f**, The V dependence of the average of $\Delta T/T$ taken at $\theta = +45°$ in the whole image. The standard deviation is shown as an error bar. The line denotes a least squares fitting line.

Supplementary Note 5 | Identification of impurities

Supplementary Figure 3 shows a scanning electron microscopy (SEM) image of a broken piece from the specimen used in the ferroaxial domain imaging of the main text. Energy dispersive x-ray (EDX) analysis revealed that the dominant gray region in the figure corresponds to $NiTiO_3$ phase while island-shaped white regions are composed of NiO impurity. The latter corresponds to dark island-shaped regions seen in the transmission optical microscopy image (Fig. 4b of the main text).

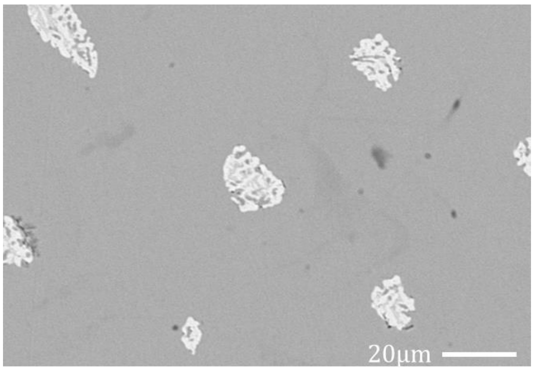

Supplementary Figure 3 | SEM image of a sample used in this study. Dominant gray and island-shaped white regions in the image correspond to $NiTiO_3$ phase and NiO impurity, respectively, which was revealed by energy dispersive x-ray (EDX) analysis. Scale bar: 20 μm.

Supplementary Note 6 | Lock-in EG measurement

We also measured EG using a lock-in technique[10] to support the results obtained by the area sensing measurement. Supplementary Figure 4 shows a schematic of the optical setup. A He-Ne laser (wavelength 632.8 nm) was used as a light source, the irradiated light was focused on a specimen by a plano-convex lens, and the transmitted light was detected by a Si amplified photodetector. The specimen with ITO transparent electrodes was placed between a polarizer and an analyzer. Both the directions of light propagation and an applied electric field were along the *c* axis (∥ **A**). The polarization configuration was set at $\theta = +45°$ or $-45°$, and the intensity of the transmitted light is expressed in the same equation with Supplementary Equation 4. In this measurement, to detect small electrogyration signals, we applied a sinusoidal voltage $V_0 \sin(\omega t)$ at a frequency of 999 Hz up to ±100 V. In this case, Supplementary Equation 4 is rewritten as;

$$I = I_0 \left(\pm\alpha V_0 \sin(\omega t) + \frac{1}{2} \right), \tag{6}$$

and the signal of $\pm\alpha V_0 I_0$ was detected with a lock-in amplifier.

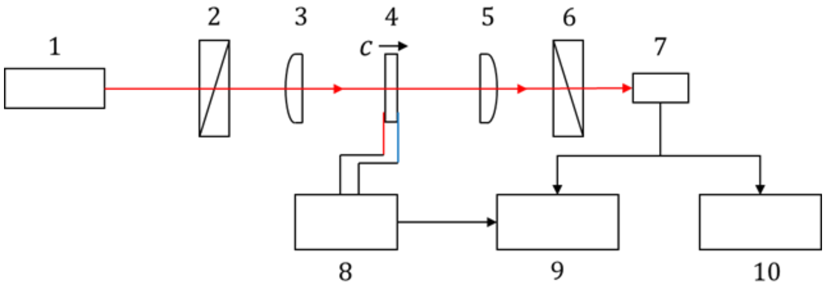

Supplementary Figure 4 | A schematic of the optical setting in the lock-in measurement. 1, He-Ne laser; 2,6, polarizers; 3,5, plano-convex lenses; 4, specimen with ITO; 7, Si amplified photodetector; 8, function generator; 9, lock-in amplifier; 10, multimeter.

Supplementary Note 7 | Domain boundaries observed by CBED

Here we show the observation of relatively thick domain boundaries in which A+ domains and A− domains overlap along the thickness direction (|| *c* axis). Supplementary Figure 5 displays a bright-field transmission electron microscope (BF-TEM) image of the area including such a domain boundary. This image was taken using the same specimen as that of Fig. 3 of the main text, but at a different area. By measuring CBED patterns at various positions in the image, spatial distributions of the domains and their boundary were determined. In the boundary area between A− and A+ domains (green colored area in Supplementary Figure 5), CBED patterns look like the superposition of those obtained at A+ and A− domains. This indicates that these two domains overlap in the boundary area. In addition, the direction of the boundary is not parallel to (110), which is different from the image shown in Fig. 3a of the main text.

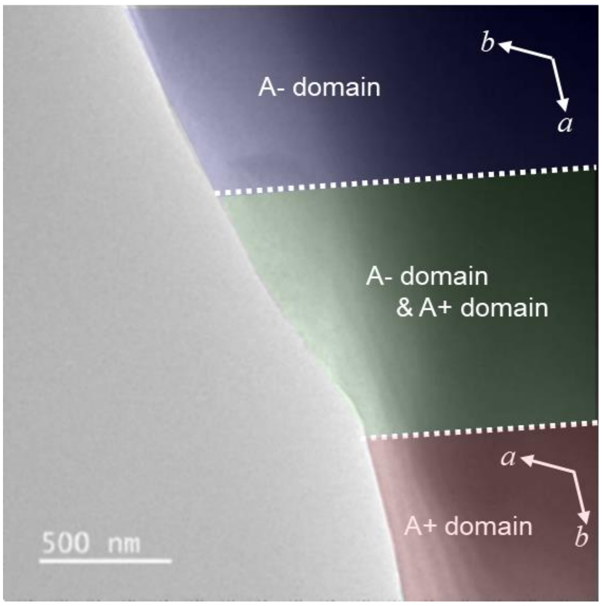

Supplementary Figure 5 | BF-TEM image of the area including a thick domain boundary. The areas of A- domain, A+ domain, and their boundary are painted in light blue, red, and green. Scale bar: 500 nm.

Supplementary References

1. Bethe, H. A. Theorie der Beugung von Elektronen an Kristallen. *Ann. Phys.* (Leipzig) **87**, 55–129 (1928).
2. Hirsch, P. B., Howie, A., Nicholson, R. B., Pashley, D. W. & Whelan, M. J. *Electron Microscopy of Thin Crystals, 2nd ed.* pp. 208-210 (Krieger, Florida; Butterworth, London, 1977).
3. Spence, J. C. H. & Zuo, J. M. *Electron Microdiffraction* (Plenum Press, New York, 1992).
4. Tsuda, K. & Tanaka, M. Refinement of crystal structural parameters using two-dimensional energy-filtered CBED patterns. *Acta Crystallogr.* **A55**, 939–954 (1999).
5. Tsuda, K., Ogata, Y., Takagi, K., Hashimoto, T. & Tanaka, M. Refinement of crystal structural parameters and charge density using convergent-beam electron diffraction - the rhombohedral phase of $LaCrO_3$. *Acta Crystallogr.* **A58**, 514–525 (2002).
6. Aroyo, M. I., Kirov, A., Capillas, C., Perez-Mato, J. M. & Wondratschek, H. Bilbao Crystallographic Server. II. Representations of crystallographic point groups and space groups. *Acta Crystallogr. Sect. A Found. Crystallogr.* **62**, 115–128 (2006).
7. Gallego, S. V., Etxebarria, J., Elcoro, L., Tasci, E. S. & Perez-Mato, J. M. Automatic calculation of symmetry-adapted tensors in magnetic and non-magnetic materials: A new tool of the bilbao crystallographic server. *Acta Crystallogr. Sect. A Found. Adv.* **75**, 438–447 (2019).
8. Aroyo, M. I. *et al.* Crystallography online: Bilbao crystallographic server. *Bulg. Chem. Commun.* **43**, 183–197 (2011).
9. Aroyo, M. I. *et al.* Bilbao Crystallographic Server I: Databases and crystallographic computing programs. *Zeitschrift fur Krist.* **221**, 15–27 (2006).
10. Novikov, M. A., Stepanov, A. A. & Khyshov, A. A. An electric sensor based on the electrogyration effect in a lead tungstate crystal. *Tech. Phys. Lett.* **43**, 372–375 (2017).
